# An Inflammatory Response-Related Gene Signature Can Predict the Prognosis and Impact the Immune Status of Lung Adenocarcinoma

**DOI:** 10.3390/cancers14235744

**Published:** 2022-11-23

**Authors:** Yubo Shi, Yingchun Zhao, Yuanyong Wang

**Affiliations:** 1 Department of Orthopaedic Surgery, Xi-Jing Hospital of the Fourth Military Medical University, Xi’an 710033, China; 2 Department of Orthopaedic Surgery, Renmin Hospital of Wuhan University, Wuhan 430064, China; 3 Department of Thoracic Surgery, Tangdu Hospital of the Fourth Military Medical University, Xi’an 710038, China

**Keywords:** inflammatory response, immune statue, tumor microenvironment, drug sensitivity, lung adenocarcinoma

## Abstract

**Simple Summary:**

Lung adenocarcinoma (LUAD) is a highly prevalent and deadly global malignancy with an annual increase in its incidence. The association of inflammation with tumor genesis and development has been of interest in recent years. However, the association of inflammatory response-related genes (IRGs) with LUAD remains unclear. In this study, we verified an inflammatory response-related gene signature to establish an independent relationship with overall survival, whose importance was in functional analysis, tumor microenvironment, drug sensitivity, and prognosis prediction in LUAD. Therefore, our study revealed the role of IRGs in tumorigenesis, especially in drug resistance, immune response, and tumor microenvironment, which is crucial in developing individualized tumor treatment.

**Abstract:**

Lung adenocarcinoma (LUAD) accounts for a cancer with high heterogeneity and poor prognostic outcome. Nonetheless, it is still unknown about the relation between inflammatory response-related genes (IRGs) and LUAD. This study used LASSO-Cox regression for establishing the multigene prognostic signature based on TCGA and the GSE31210 cohorts. In addition, gene set enrichment analysis (GSEA) was performed for GO and KEGG analyses. By contrast, single-sample GSEA (ssGSEA) investigated immune cell infiltration scores as well as the immune pathway activity. We also conducted qRT-PCR and IHC to evaluate prognostic gene expression at protein and mRNA levels within LUAD and adjacent healthy samples. As a result, a novel prognostic signature involving 10 IRGs was identified. Furthermore, the signature has been validated as being important in functional analysis, TME, drug sensitivity, and prognosis prediction in LUAD. Moreover, prognostic genes showed significant expression at protein and mRNA levels in LUAD compared with normal samples. The signature involving 10 IRGs could potentially predict LUAD prognosis.

## 1. Introduction

Lung cancer (LC) is a highly prevalent and deadly global malignancy with an annual increase in its incidence [1]. LC is classified as non-small cell lung cancer (NSCLC) or small cell lung cancer (SCLC) based on pathological features, with the former occupying 85–90% of all the LC cases [2]. Moreover, lung adenocarcinoma (LUAD) is the frequently seen NSCLC subtype. Unfortunately, LUAD patients are diagnosed at intermediate or advanced stages due to a lack of sensitive early screening tools [3,4]. Thus, understanding biological mechanisms and discovering novel biomarkers associated with LUAD is essential to improve the prognostic assessment of patients.

The association of inflammation with malignancy has been extensively studied [5]. In addition, the role of inflammation during tumor genesis and development has been of interest in recent years. Inflammation is like a double-edged sword, promoting or suppressing cancer occurrence [6]. The correlation between inflammation and cancer can be explored using commonly used inflammatory markers. For example, the Glasgow score evaluating C-reactive protein (CRP) and albumin (Alb) projects prognostic performance among tumor cases [7]. Evaluation of clinically inflammatory biomarkers in new LC cases, including lymphocyte-monocyte ratio (LMR), platelet-lymphocyte ratio (PLR), and mesangial cell ratio (MCR), could independently predict overall survival (OS) [8]. Studies support inflammation-based prognostic scores for cancers by combining various acute-phase proteins. Apart from serum markers, several inflammatory response-related genes (IRGs) can be utilized in forecasting tumor progression [9,10]. However, the association of IRGs with LUAD remains unclear.

In this study, the clinical and mRNA expression data of LUAD patients were downloaded from freely accessible databases. Afterwards, a prognostic nomogram based on inflammatory response-related differentially expressed genes (DEGs) was built according to TCGA cohort, while the GEO cohort was adopted for validation. Subsequently, we conducted functional annotation for investigating associated mechanisms. Additionally, relations between gene prognostic significance and tumor stemness, tumor chemoresistance, as well as immune infiltrates were analyzed. Finally, predictive gene protein and mRNA levels were detected in LUAD samples and compared with normal samples using qRT-PCR and immunohistochemistry (IHC).

## 2. Materials and Methods

### 2.1. Data Extraction (TCGA-LUAD and GEO(GSE31210) Cohorts)

The clinical and RNA-sequencing (RNA-seq) data were downloaded from the public GEO (http://www.ncbi.nlm.nih.gov/geo/ (accessed on 25 March 2019)) (*n* = 226) and TCGA (https://www.cancer.gov/tcga (accessed on 28 September 2022)) (*n* = 522) datasets through following the publication guidelines and overall data access policies. In addition, we obtained 200 IRGs from the Molecular Signatures Database (Supplementary Table S1).

### 2.2. Establishment and Verification of the Prognosis Model of IRGs

We compared DEGs in cancer patients with non-carcinoma samples using the “limma” function in the R package upon the thresholds of fold change (FC) > two and false discovery rate (FDR) < 0.05 from TCGA cohort. Then, we conducted univariate Cox regression for evaluating the prognostic IRGs. We established the prognosis model through LASSO-penalized Cox regression analysis to minimize the overfitting risk [11]. Using the “glmnet” function in the R package, we shrunk variables by the LASSO algorithm to rigorously equalize specific regression coefficients to zero, yielding an interpretable model. We evaluated the penalty parameter (λ) of the prognosis model based on minimum criteria using 10-fold cross-validation. Patients’ risk scores were identified based on levels of IRGs and the matching regression coefficient was evaluated according to score = e^sum (every gene’s level × relevant c^°^efficient)^. The cases were characterized as either low- or high-risk groups following median risk score. Finally, we conducted t-SNE and PCA analyses using the “ggplot2” and “Rtsne” functions to explore various group distributions. R package “survminer” function was adopted to evaluate both groups’ OS. Furthermore, we conducted a time-dependent ROC (t-ROC) survival analysis to assess the predictive significance of our prognosis model. Moreover, we performed univariate/multivariate Cox regression to analyze whether the prognosis model independently predicted prognosis [9].

### 2.3. Functional Annotation

We conducted Gene Ontology (GO) and Kyoto Encyclopedia of Genes and Genomes (KEGG) analyses through gene set enrichment analysis (GSEA) based on the DEGs between both groups by using the GSEA software 4.1. In addition, we employed single-sample GSEA (ssGSEA) to compare 16 immune cell infiltration scores and 13 immune pathway activities between low- and high-risk cohorts with “GSVA” function in the R package.

### 2.4. Tumor Microenvironment (TME) and Immune Response Analysis

Immune and stromal scores evaluated immune/stromal cell infiltration levels within LUAD tissues [12]. Associations of the above scores with the risk scores were determined through Spearman’s correlation. In contrast, a two-way ANOVA analysis detected the association of risk score with immune infiltration subtypes. Moreover, the epigenetic and transcriptomic data of TCGA specimens were employed to quantify cancer stem cell (CSC)-like characteristics. Finally, we also conducted Spearman’s correlation test to analyze the relationship of risk score with tumor stemness.

### 2.5. ChemoSensitivity

By using the CellMiner interface (https://discover.nci.nih.gov/cellminer/ (accessed on 25 March 2019)), we searched the NCI-60 database, having 60 diverse tumor cell types from nine various cancers. Pearson’s correlation analysis was conducted for assessing the relations of prognostic gene levels with drug sensitivity. In addition, correlation analysis was performed for determining whether 263 drugs approved by FDA or in clinical trials were effective (Supplementary Table S2).

### 2.6. RNA Isolation and qRT-PCR Analysis

Thirty-four paired LUAD and normal tissues were obtained from the Tangdu Hospital of Fourth Military Medical University. The Ethics Committee of Tangdu Hospital approved the study. Then, qRT-PCR evaluated mRNA levels of IRGs in LUAD and corresponding healthy samples. After that, we collected total RNA from clinical samples with TRIzol reagent (Invitrogen, Waltham, MA, USA), and cDNA was prepared using a kit (Takara, Dalian, China). Finally, SYBR Green PCR Kit (Takara, Dalian, China) was used for qPCR, with GAPDH as the endogenous reference. qRT-PCR conditions were set according to the manufacturer’s instructions. QuantStudio Dx Real-Time PCR Instrument (Thermo Fisher Scientific, Waltham, WA, USA) was used for qRT-PCR. All the primers are shown in Supplementary Table S3.

### 2.7. Immunohistochemistry (IHC)

The IHC experiment detected protein levels of IRGs in LUAD and corresponding normal tissues. First, each sample was subjected to 10% formalin fixation, paraffin embedding, and processing up to 4-µm consecutive sections. After that, the sections were treated with methanol, followed by BSA incubation and primary antibody staining. Then, we stained the samples with a secondary antibody after washing with PBS. Finally, a microscope was utilized for observing and photographing every section. This study acquired primary antibodies in Abcam (Supplementary Table S4).

### 2.8. Statistical Analysis

We compared DEGs in cancer samples with surrounding samples using the Wilcoxon test. The Chi-squared test compared the percentage differences. In addition, the Mann-Whitney test compared immune cell and immune pathway ssGSEA scores of low-versus high-risk groups, and the *p*-values were normalized using the BH approach. Different OS scores were compared among diverse cancer groups using Kaplan-Meier (K-M) analysis. Predicting factors for OS were determined by univariate/multivariate Cox regression. Relationships between the risk score of the prognosis model and immune/stromal/stemness scores and drug sensitivity were analyzed using Pearson’s correlation analysis. *p* < 0.05 (two-tailed) was accepted as the standard for statistical significance.

## 3. Results

Table 1 shows the study flowchart with 552 TCGA-LUAD cases and 226 LUAD cases in the GSE31210 dataset. Table 1 represents the clinical characteristics of patients.

### 3.1. Discovery of Prognostic IRGs from TCGA Dataset

**Figure 1:**
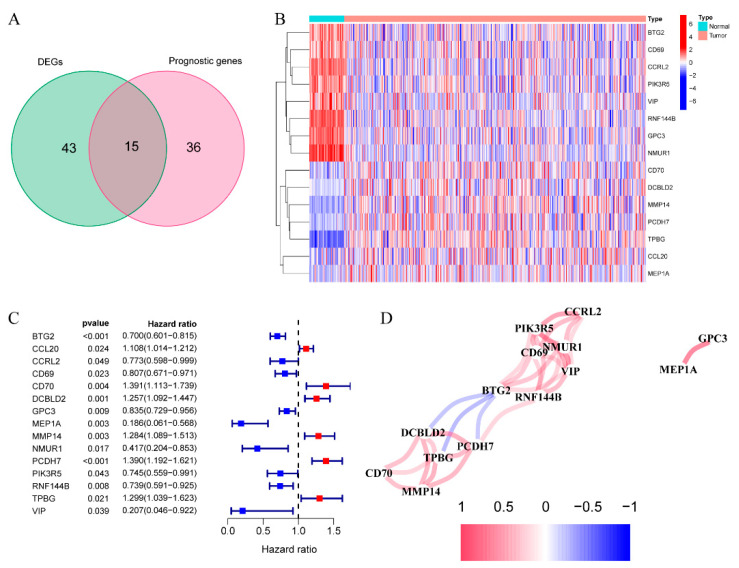
Discovery of the 15 IRGs from TCGA dataset. (**A**) DEGs in LUAD samples compared with the non-carcinoma samples. (**B**) The levels of 15 candidate genes in LUAD samples compared with non-carcinoma samples. (**C**) Association of the 15 putative gene levels with OS. (**D**) Putative gene correlation network.

We observed that 58 IRGs showed differential expression between cancer and healthy samples, which was collected from (Genotype-Tissue Expression) GTEx datasets. Based on the univariate Cox regression, 15 were related to OS (Figure 1A,B). The predictors included 15 IRGs for PCDH7 gene, with a risk ratio of 1.39 (95% CI = 1.192–1.621, *p* < 0.001, Figure 1C). Figure 1D represents the association of the genes mentioned above.

### 3.2. Establishment of the Prognosis Model for TCGA Dataset

**Figure 2:**
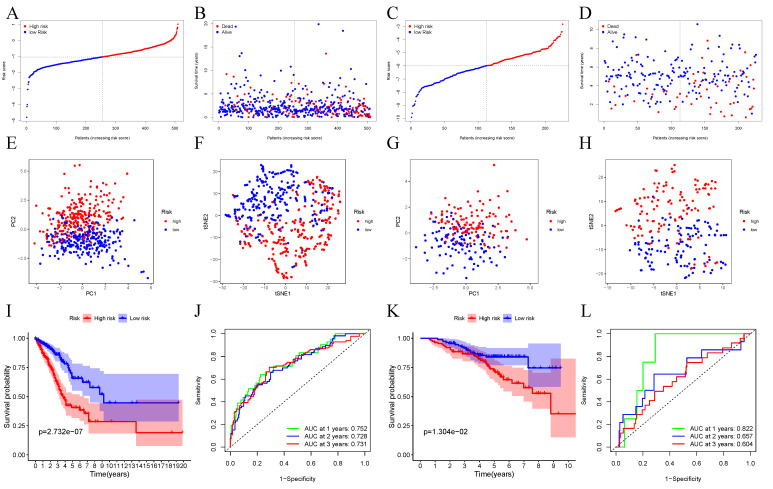
Validation of our 10-gene signature based on TCGA and GEO datasets. TCGA (**A**,**B**,**E**,**F**,**I**,**J**) and GEO (**C**,**D**,**G**,**H**,**K**,**L**) datasets. (**A**,**C**) Median risk scores. (**B**,**D**) OS distribution. (**E**,**G**) PCA plot. (**F**,**H**) t-SNE analysis. (**I**,**K**) K-M survival analysis of OS. (**J**,**L**) AUC value of t-ROC curves for OS.

We employed the LASSO-Cox regression to investigate 15 gene expression levels. Based on the best λ, a marker of 10 genes was identified (Supplementary Figure S1). We determined the risk score below: score = 0.189 ∗ CD70 expression − 0.114 ∗ CD69 expression + 0.097 ∗ CCL20 expression + 0.0338 ∗ DCBLD2 expression − 0.777 ∗ MEP1A expression + 0.031 ∗ MMP14 expression + 0.198 ∗ PCDH7 expression − 0.093 ∗ PIK3R5 expression − 0.156 ∗ RNF144B expression − 0.194 ∗ BTG2 expression. We divided cases into two groups according to the median threshold and divided cases into two groups (Figure 2A). In TCGA cohort, the high-risk group was tightly associated with the later TNM stage (Table 2). The scatter plots showed that high-risk patients had a shorter OS than low-risk ones (Figure 2B). According to t-SNE and PCA analyses, all cases were classified into two groups (Figure 2E,F). Additionally, Kaplan-Meier (K-M) curves demonstrated that the high-risk group was associated with a markedly shorter OS than low-risk ones (Figure 2I). The t-ROC curve analysis investigated the predicting ability of the prognostic model, and the 1-, 2- and 3-year area under the curve (AUC) values reached 0.752, 0.728, and 0.731, respectively (Figure 2J). In line with the best threshold levels of respective prognostic genes, we conducted a survival analysis to investigate the relation of prognostic genes with prognosis. The results showed that up-regulation of the above genes predicted dismal OS (Supplementary Figure S2A–H).

### 3.3. Verification of 10-Gene Signature Using GEO Dataset

In order to confirm the validity of the model constructed based on TCGA cohort, the GEO cases were divided as low- or high-risk groups in accordance with median TCGA samples (Figure 2C). Based on PCA and t-SNE analyses, patients were dispersedly distributed in two subgroups, thus conforming to analysis based on TCGA cohort (Figure 2G,H). High-risk patients showed an increased premature death risk (Figure 2D) and the decreased survival time (Figure 2K). In addition, the 1-, 2- and 3-year AUC values for the 10-gene signatures were 0.822, 0.657, and 0.604, respectively (Figure 2L).

### 3.4. The Ability of Our 10-Gene Signature to Independently Predict Prognosis

**Figure 3:**
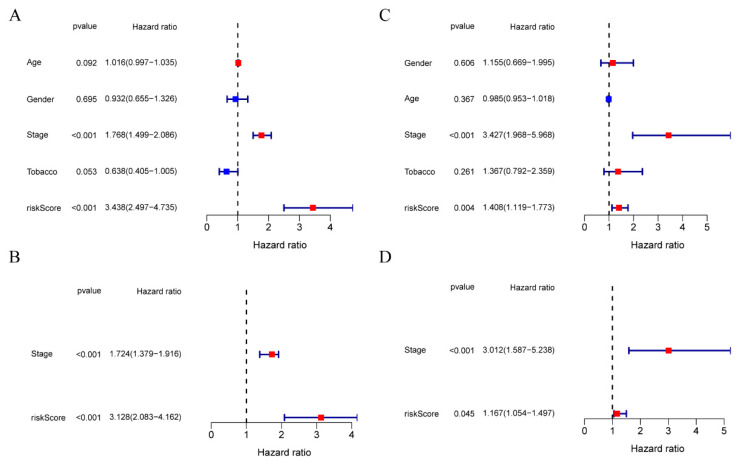
Comparison of clinicopathological features and risk score accuracy in prognosis prediction. TCGA (**A**,**B**) and GEO (**C**,**D**) cohorts. (**A**,**C**) OS-associated factors selected through univariate Cox regression. (**B**,**D**) OS-associated elements selected through multivariate Cox regression.

Univariate/multivariate Cox regression was performed on each variable for investigating the independent predicting ability of risk score for OS. According to univariate regression, risk scores of TCGA and the GEO datasets were markedly associated with OS (GEO dataset: HR = 1.408, 95% CI = 1.119–1.773, *p* = 0.004; TCGA dataset: HR = 3.438, 95% CI = 2.497–4.735, *p* < 0.001) (Figure 3A,C). Subsequently, our risk score independently predicted OS based on the multivariate Cox analysis (GEO dataset: HR = 1.167, 95% CI = 1.054–1.497, *p* = 0.045; TCGA dataset: HR = 3.128, 95% CI = 2.083–4.162, *p* < 0.001) (Figure 3B,D).

### 3.5. Risk Score of Prognosis Model and Clinical Characteristics

**Figure 4:**
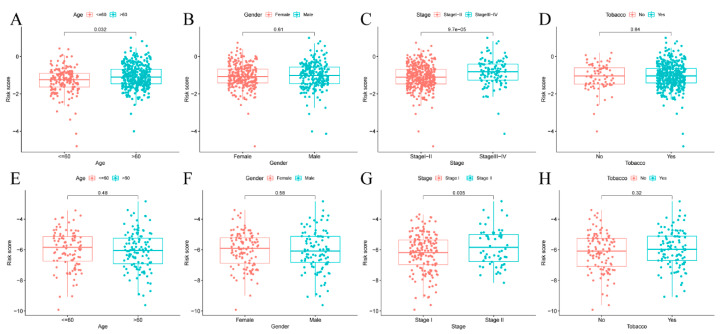
The risk scores of diverse groups were evaluated based on distinct clinical features. TCGA (**A**–**D**) and GEO (**E**–**H**) datasets. (**A**,**E**) Age. (**B**,**F**) Sex. (**C**,**G**) Tumor stage. (**D**,**H**) Tobacco.

This study observed that the risks scores were not significantly different between patients aged ≤60 and >60 years from the GEO dataset after examining the relationship between risk scores and clinical features among LUAD cases. In contrast, risk scores were significantly higher in patients aged > 60 years than ≤60 years from TCGA dataset (*p* = 0.032) (Figure 4A,E). In addition, stage I-II patients had significantly lower risk scores than stage III-IV patients belonging to TCGA and the GEO datasets (Figure 4C,G). Additionally, there was no significant difference in gender (Figure 4B,F) and tobacco use between the two groups (Figure 4D,H).

### 3.6. Immune Status and TME Analyses

**Figure 5:**
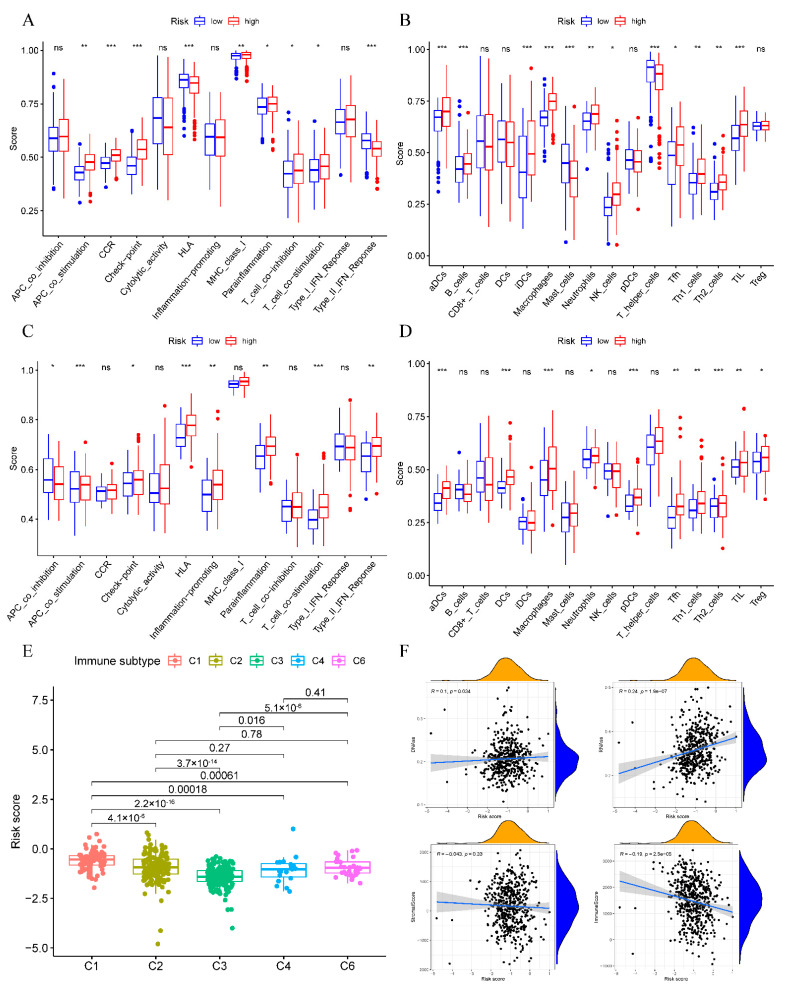
Immune status of diverse risk groups and the relation of the risk score with TME. TCGA (**A**,**B**) and GEO (**C**,**D**) datasets. (**A**,**C**) Boxplots showed 13 immune-associated functions and (**B**,**D**) Scores of 16 immune cells. (**E**) Risk scores compared among diverse immune infiltration types. (**F**) Association of the risk score with DNAss, RNAss, Immune/Stromal Scores. * *p* < 0.05; ** *p* < 0.01; *** *p* < 0.001; ns, not significant.

We conducted ssGSEA to quantify enrichment scores for various immune cells, pathways, and functions to investigate the association of risk scores with immune status. High-risk patients had elevated scores of CCR, checkpoint, inflammation-promoting activity, and type II IFN response activity than low-risk patients. In addition, we evaluated the antigen presentation process involving aDCs and iDCs, among high-risk patients (Figure 5A,B). Moreover, high-risk patients had increased proportions of Th1, Th2, and Tfh cells, T cell co-stimulation, and T cell co-inhibition compared to low-risk patients. These results were similar in TCGA and the GEO database groups (Figure 5C,D).

We further analyzed the link of risk score with immune infiltration to explore the association of risk score with immune components. We constructed six different immune infiltrations, viz., C1 (wound healing), C2 (INF-γ dominance), C3 (inflammation), C4 (lymphocyte depletion), C5 (immune quieting), and C6 (TGF-β dominance) within human tumors [13]. Since no LUAD sample matched the C5 subtype, the study ruled it out. As a result, high-risk scores were significantly associated with C1, while low-risk scores were substantially associated with C3 (Figure 5E).

The mRNA expression-based RNA stemness score (RNAss) and the DNA methylation pattern-based DNA stemness (DNAss) score were employed to measure tumor stemness [14]. In addition, TME was assessed by immune and stromal scores. We conducted a correlation analysis to investigate the association of risk scores with tumor stemness and TME. Thus, the risk score showed a significant correlation with DNAss and RNAss, and a negative association with immune score (Figure 5F).

**Figure 6:**
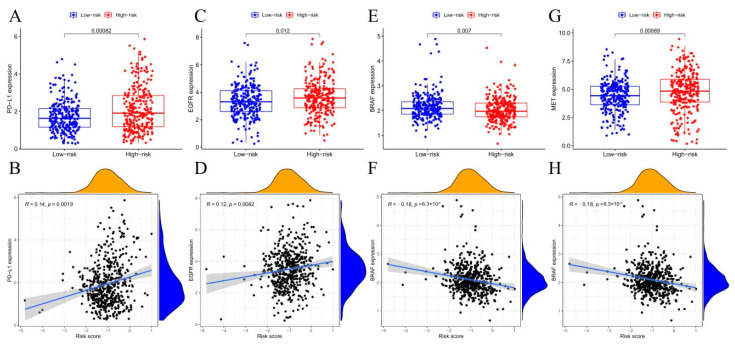
PD-1, EGFR, BRAF, and MET levels compared in the two risk groups and correlations of the risk score with PD-1, EGFR, BRAF, and MET levels. (**A**,**B**) PD-1. (**C**,**D**) EGFR. (**E**,**F**) BRAF. (**G**,**H**) MET.

In cancer immune evasion, immune checkpoints such as PD-L1 are critical for customized treatment. PD-1 expression levels were considerably elevated among high-risk patients than low-risk patients and negatively correlated with risk scores (Figure 6A,B). In addition, the expression of tumor drug resistance genes, EGFR, and MET were remarkably higher among the high-risk group than low-risk patients, positively related to risk score (Figure 6C,D,G,H). However, BRAF was the opposite (Figure 6E,F).

### 3.7. Biological Function and Pathway Analyses

**Figure 7:**
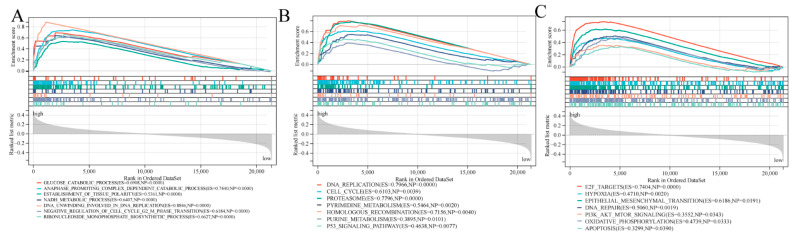
GSEA on biological roles as well as pathways. (**A**) GO analysis. (**B**) KEGG analysis. (**C**) Hallmark gene set.

We conducted GO functional annotation and KEGG analyses on both groups using GSEA. GO functional annotation revealed that the glucose catabolic process was significantly associated with the high-risk cohort (Figure 7A). In addition, the high-risk group was correlated with several cancer-associated pathways, like cell cycle, DNA replication, and P53 signaling pathways. Moreover, purine metabolism and oxidative phosphorylation pathways were related to inflammatory responses (Figure 7B). The GSEA based on Hallmark gene sets revealed that epithelial-mesenchymal transition pathway, PI3K-AKT-mTOR-Signaling, and oxidative-phosphorylation were statistically significant programs (Figure 7C).

### 3.8. Prognostic Gene Levels and Drug Sensitivity of Cancer Cells

**Figure 8:**
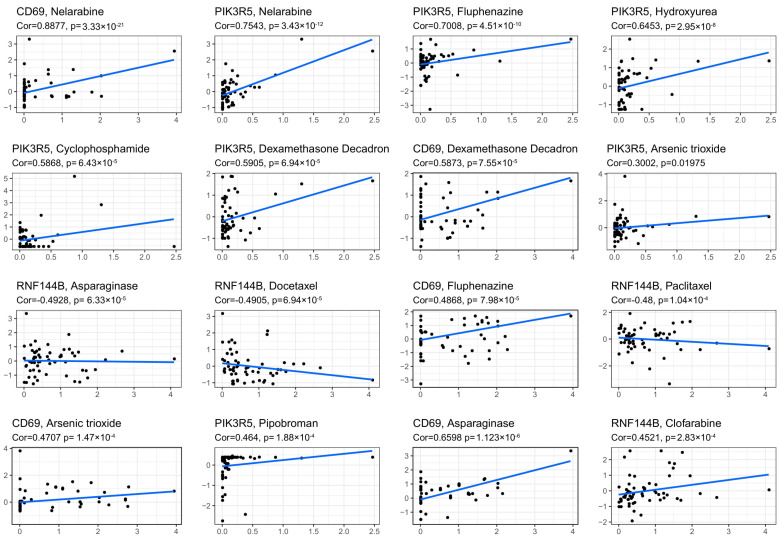
Scatter plot showing the association of levels of prognostic genes with sensitivity to drugs.

The association between the prognostic gene levels and drug sensitivity of NCI-60 cells was also investigated. We found that the prognostic genes were associated with some chemotherapeutic sensitivities (Figure 8). For example, up-regulated CD69 and PIK3R5 were associated with higher tumor cell sensitivity with various chemotherapeutics like nelarabine, fluphenazine, and dexamethasone decadron. In contrast, RNF144B up-regulation correlated with tumor cell resistance against docetaxel and asparaginase.

### 3.9. Validation of mRNA and Protein Expression of Prognostic Genes

**Figure 9:**
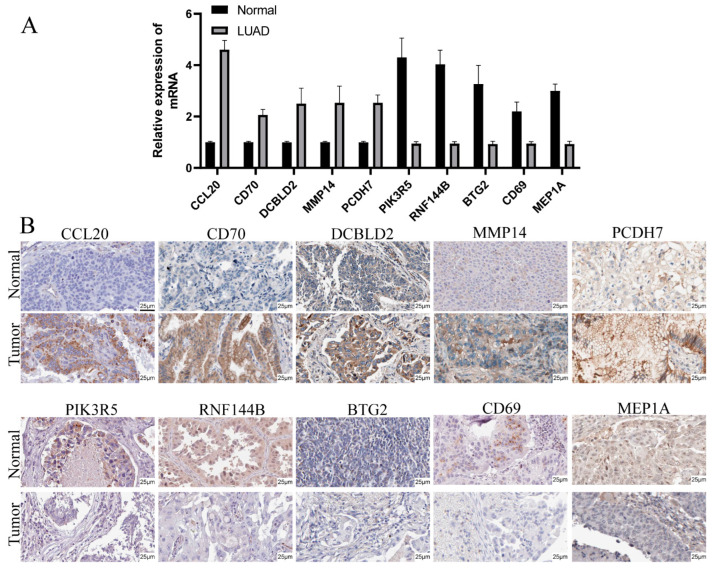
Prognostic gene protein and mRNA levels in LUAD compared with healthy samples. (**A**) mRNA levels were analyzed through qRT-RCR. (**B**) Protein levels were analyzed through IHC.

We conducted qRT-PCR and IHC for evaluating 10-prognostic gene protein and mRNA levels in LUAD and compared them with healthy samples. The qRT-PCR analysis demonstrated that CCL20, CD70, PCDH7, DCBLD2, and MMP14 genes were remarkably elevated within LUAD samples than adjacent non-tumorous tissues, where PIK3R5, RNF144B, BTG2, CD69, and MEP1A genes were the opposite (Figure 9A). In addition, IHC results were consistent with data from qRT-PCR. The expression of CCL20, CD70, PCDH7, DCBLD2, and MMP14 genes were remarkably more elevated within LUAD samples than adjacent non-tumorous tissues, where PIK3R5, RNF144B, BTG2, CD69, and MEP1A genes were the opposite (Figure 9B).

## 4. Discussion

Due to sequencing technologies and precision medicine advances, significant progress has been achieved in treating LUAD, but LC remains a highly lethal tumor worldwide. It is difficult to diagnose and predict LUAD prognosis as reliable indicators are lacking. Therefore, a new strategy to analyze and predict LUAD prognosis is quintessential. DNA methylation, tumor DNA, miRNA, and circulating tumor cells were previously reported to help depict the prognostic significance of LUAD [15,16]. In addition, serum biomarkers associated with inflammatory response, like LMR, MCR, and PLR also showed higher accuracy in predicting LUAD prognosis [17]. It is worth noting that the signature in our research overlaps quite a bit with the gene signature reported by Song et al. However, there are also some differences between these two signatures. The t-ROC curve analysis was performed to investigate the predicting ability of the prognostic model. The 1-, 2- and 3-year area under the curve (AUC) values reached 0.752, 0.728, and 0.731 in our signature, respectively, whereas the 1-, 2- and 3-year AUC values in Song et al.’s study was 0.719, 0.705 and 0.701. Thus, the predicting ability of our prognostic model was higher than that of their study [18]. Research has shown that hypoxia-related, ferroptosis-related, and immune-related gene signatures predict OS at three years, similar to our results [19,20,21]. In addition to the excellent ability to predict LUAD prognosis, our constructed IRG signature displayed superiorities to those gene signatures mentioned above and distinguished tumor resistance and immune checkpoint genes within high-or low-expression groups. Moreover, risk scores are associated with specific chemotherapeutic resistance. This work assessed prognostic signature gene expression using high-throughput sequencing (HTS), which is the standard technology for yielding precise analysis.

We explored the expression of 200 IRGs within LUAD samples and their association with OS. In TCGA dataset, 58 DEGs were found, and 15 were correlated with OS. Subsequently, we underwent LASSO regression to develop the prognosis model, including 10 IRGs. We divided cases as high- or low-risk groups based on median risk scores. Our results indicated that high-risk patients had reduced OS and advanced tumor grade. In addition, we verified the risk score to predict prognosis by independent predictive analysis.

In this study, a prognosis model containing 10 IRGs was constructed. CCL20, CD70, DCBLD2, PCDH7, and MMP14 expression were remarkably elevated within the LUAD samples, predicting the dismal survival. On the other hand, PIK3R5, RNF144B, CD69, BTG2, and MEP1A had contrary results. CCL20 belongs to the chemokine family and promotes lung cancer cell migration and proliferation [22]. CD70 belongs to the tumor necrosis factor (TNF) family which is critical in cancer immunotherapy [23]. DCBLD2 is localized within the cytosol and on the plasma membrane, mediating cisplatin-induced metastasis in LUAD [24]. MMP14, which belongs to the matrix metalloproteinase (MMP) family, has an essential effect on tumor migration. PCDH7 belongs to the cadherin superfamily and is involved in cancer stem cell oncogenesis. PIK3R5 is a component of PI3 kinase and sustains stemness by the PI3K-AKT pathway [25]. RNF144B is an E3-ubiquitin ligase and could be a new candidate for targeted treatment [26]. BTG2 is recognized as a tumor suppressor, and its overexpression has been revealed to suppress LC cell proliferation [27]. MEP1A is a member of the astaxanthin family and critically affects forecasting cancer patient prognosis [28].

Due to there being fewer studies on these genes, it remains unclear whether they could impact LUAD survival by the inflammatory response. Cancer-associated signaling pathways like the P53 pathway showed significant enrichment using GSEA analysis. In addition, sustained pathway activation is related to LUAD, which could behave as novel therapeutic targets [29]. Pathways related to inflammation, such as purine metabolism and oxidative phosphorylation, showed significant enrichment within a high-risk cohort, indicating a close relationship between tumor progression and inflammatory response. Immune checkpoints like PD-1 inhibit the antitumor immune response of T cells, and immune checkpoint inhibitors (ICIs) have attained significant advances in treating LUAD [30,31]. Our results have shown that high-risk patients had increased immune checkpoint scores than low-risk patients, and the risk scores were positively correlated with PD-1 expression. Therefore, this prognostic model could guide treatment decision-making by predicting ICI expression. Moreover, high-risk patients with higher Th1 cells, Th2 cells, Tfh cells, T cell co-stimulation, and T cell co-inhibition activities suggest immune regulation impairment, which may cause poor prognosis in the high-risk patient group.

Cancer biology has shifted from the “tumor cell-focus” perspective to the concept of embedded tumor cells within the stromal cells network that contains fibroblasts, inflammatory immune cells, and vascular cells. TME is made up of these cells [6]. CSCs have been obtained by dedifferentiation of non-stem cells, long-lived stem cells, and progenitor cells [14]. CSCs could promote tumor development due to their ability to self-renew and invade, representing the primary reason behind drug resistance after treatment [32]. In addition, associations of TME, risk scores, with CSCs were examined, as a result, risk score was significantly related to RNAss, DNAss, immune and stromal scores.

The association of the prognostic gene levels with drug resistance in NCI-60 cells was also investigated. As a result, the up-regulation of RNF144B predicted cancer cell resistance against docetaxel and asparaginase. Moreover, certain prognostic genes are related to enhanced drug sensitivity. For instance, higher expression of CD69 and PIK3R5 was associated with enhanced sensitivity of tumor cells toward multiple chemotherapeutics such as nelarabine, fluphenazine, and dexamethasone decadron. Thus, specific prognostic genes could be the therapeutic targets for overcoming drug sensitivity or resistance.

## 5. Conclusions

The new prognosis model involving ten IRGs was identified in our study. Furthermore, based on the GEO and TCGA datasets, we verified our constructed signature to establish an independent relationship with OS, whose importance was in functional analysis, TME, drug sensitivity, and prognosis prediction in LUAD. Therefore, our study revealed the role of IRGs in tumorigenesis, especially in drug resistance, immune response, and TME, which is crucial in developing individualized tumor treatments.

## Figures and Tables

**Table 1:** Clinical characteristics of the LUAD patients.

	TCGA-LUAD	GSE31210
Cases	522	226
Age (Median, range)	65 (33–88)	59 (30–76)
Gender		
Female	280 (53.64%)	121 (53.54%)
Male	242 (43.36%)	105 (46.46%)
Tabacco		
Yes	443 (84.86%)	111 (49.12%)
No	79 (15.14%)	115 (50.88%)
Stage		
I	282 (54.02%)	168 (74.34%)
II	127 (24.33%)	58 (25.66%)
III	87 (16.67%)	NA
IV	26 (4.98%)	NA
Survival Status		
Alive	384 (73.56%)	174 (77%)
Dead	138 (26.44)	52 (23%)

**Table 2:** Baseline characteristics of the patients in different risk groups.

Characteristics	TCGA-LUAD Cohort			GSE31210 Cohort		
	High Risk	Low Risk	*p* Value	High Risk	Low Risk	*p* Value
Age			0.008			0.287
≤60 year	95	67		58	50	
>60 year	160	188		55	63	
Gender			0.477			0.23
Female	134	142		65	56	
Male	121	113		48	57	
Stage			<0.0001			0.034
I	117	158		71	85	
II	65	59		35	28	
III	62	24		NA	NA	
IV	11	14		NA	NA	
Tabacco			1			0.506
Yes	216	216		58	53	
No	39	39		55	60	

## Data Availability

The raw data supporting the conclusions of this article will be made available by the authors, without undue reservation.

## Supplementary Materials

The following supporting information can be downloaded at: https://www.mdpi.com/article/10.3390/cancers14235744/s1, Figure S1: Constructed an 8-gene signature in the TCGA cohort; Figure S2: Survival analysis of prognostic genes according to the optimal cut-off expression value. TCGA cohort; Table S1: The 200 inflammatory response-related genes; Table S2: The 263 chemotherapy drugs of FDA approved or on clinical trials; Table S3: The primers of ten-prognostic genes; Table S4: Protein antibody stock number.
